# Essential Oil Composition of Seven Bulgarian *Hypericum* Species and Its Potential as a Biopesticide

**DOI:** 10.3390/plants12040923

**Published:** 2023-02-17

**Authors:** Ivanka Semerdjieva, Valtcho D. Zheljazkov, Ivayla Dincheva, Neshka Piperkova, Vasilina Maneva, Charles L. Cantrell, Tess Astatkie, Albena Stoyanova, Tanya Ivanova

**Affiliations:** 1 Department of Botany and Agrometeorology, Agricultural University, 4000 Plovdiv, Bulgaria; 2 Department of Plant and Fungal Diversity and Resources, Institute of Biodiversity and Ecosystem Research, Bulgarian Academy of Sciences, 1013 Sofia, Bulgaria; 3 Crop and Soil Science Department, Oregon State University, Corvallis, OR 97331, USA; 4 Plant Genetic Research Group, AgroBioInstitute, Agricultural Academy, 1164 Sofia, Bulgaria; 5 Department of Phytopathology, Agricultural University, 4000 Plovdiv, Bulgaria; 6 Plant Protection and Technology Department, Institute of Agriculture, Agricultural Academy, 8400 Karnobat, Bulgaria; 7 Natural Products Utilization Research Unit, USDA-ARS, University, MS 38677, USA; 8 Faculty of Agriculture, Dalhousie University, Truro, NS B2N 5E3, Canada; 9 Department of Technology of Fats, Essential Oils, Perfumery and Cosmetics, University of Food Technologies, 4002 Plovdiv, Bulgaria

**Keywords:** *Hypericum*, Bulgaria, repellent, insecticidal, antifungal, essential oil

## Abstract

*Hypericum* species and especially *H. perforatum* L. are well known for their therapeutic applications. The present study assessed the essential oil (EO) composition, and antifungal and aphid suppression activity of seven Bulgarian *Hypericum* species. The EOs were analyzed by GC–MS–FID. Two experiments were conducted. In the first experiment, *H. perforatum*, *H. maculatum*, and *H. hirsutum* were used. Additionally, the EO composition of *H. perforatum* extracted via hydrodistillation (ClevA) and via commercial steam distillation (Com) were compared. The second experiment compared the EOs of *H. perforatum*, *H. cerastoides*, *H. rumeliacum*, *H. montbretii*, and *H. calycinum* (flowers and leaves) extracted via hydrodistillation and collected with *n*-hexane. Overall, the EO constituents belonged to four classes, namely alkanes, monoterpenes, sesquiterpenes, and fatty acids. The main class for compounds in *H. maculatum* and *H. perforatum* (section *Hypericum*) were sesquiterpenes for both experiments except for *H. perforatum* (Com). *Hypericum montbretii* (section *Drosocarpium*) EO had monoterpenes (38.09%) and sesquiterpenes (37.09%) as major groups, while *H. hirsutum* EO (section *Taeniocarpium*) contained predominately alkanes (67.19%). *Hypericum hirsutum* EO contained cedrol (5.04%), found for the first time in *Hypericum* species. Fatty acids were the main compounds in *H. cerastoides* (section *Campylopus*), while monoterpenes were the most abundant class in *H. rumeliacum* and *H. calycinum* EOs. *α*-Pinene and germacrene D were the major EO constituents of all analyzed *Hypericum* species except for *H. hirsutum* and *H. cerastoides. Hypericum perforatum* EO (Com) had significant repellent and insecticidal activity against two aphid species, *Rhopalosiphum padi* (Bird Cherry-oat aphid) and *Sitobion avenae* (English grain aphid) at concentrations of 0%, 1%, 2.5%, 3.5%, 4.5%, and 5%. The tested EOs did not show significant activity against selected economically important agricultural fungal pathogens *Fusarium* spp., *Botrytis cinerea*, *Colletotrichum* spp., *Rhizoctonia solani*, and *Aspergillus* sp. The EO of the *Hypericum* species found in the Bulgarian flora could be utilized for the development of new biopesticides for aphid control.

## 1. Introduction

The genus *Hypericum* (Hypericaceae/Guttiferae) is represented by 61 species in 17 sections in the European flora [1]. Although there are 22 species of the genus *Hypericum* in Bulgaria, five of which are Bulgarian or Balkan endemics, rare, and protected species, *H. perforatum* L. has been the most widely studied primarily because it is the most widespread [2]. The extracts from *H. perforatum* have a wide range of biological activities such as antiviral, wound healing, antioxidant, and antimicrobial activities [3,4,5,6,7]. The pharmacological purpose of the species’ preparations is to treat depressed moods, nervousness, anxiety, wounds, inflammation, neuralgia, malaria, headache, bedwetting, and mild-to-moderate depression [8,9,10]. The other species of the genus *Hypericum* have more limited uses in traditional medicine, although some are widely used as ornamentals [11,12].

Naphthodianthrones (e.g., hypericin and pseudohypericin), flavonol glycosides (e.g., isoquercitrin and hyperoside), biflavonoids (e.g., amentoflavone), phloroglucinol derivatives, and xanthones have been the most studied compounds of *Hypericum* species [13,14,15,16,17]. In general, studies on the essential oil (EO) composition of *Hypericum* species are sparse, and the published results on the chemical profile vary widely [18] (Table 1). The reasons for the differences in the reported EO composition may be due to the different methods of EO extraction, such as steam distillation [7,17], hydrodistillation by Clevenger-type apparatus [15], and micro-simultaneous distillation–extraction [19,20] (Table 1). Furthermore, it is also reported that changes in the EO composition of *Hypericum* species are influenced by various other factors such as seasonal variation, geographic distribution, phenological cycle, and the plant part in which the EO is accumulated [2,21]. 

Plant essential oils (EOs) have been known to possess biopesticidal activity against pests and phytopathogens [3,4]. Some of the advantages of the EOs as biopesticides are their effectiveness, low toxicity, and multiple mechanisms of action [22]. *Hypericum perforatum* is widely distributed but there is insufficient information on the biopesticide activity of its EO. Only two articles were found on the antifungal activity of *Hypericum hyssopifolium* subsp*. elongatum* var*. elongatum* and *H. heterophyllum* Vent. [3,4]. The lack of research data on *Hypericum* EOs as biopesticides is the reason for the need to assess their potential as ingredients when developing new products. Some of the challenges that would need to be addressed in future research include (1) the development of new technologies for the manufacture of the products with EOs (e.g., microencapsulation); (2) simplification of the complex and costly biopesticide authorization requirements; (3) cultivation of plants and optimization of extraction procedures; and (4) achieving homogeneous chemical composition [22]. There are scarce data on the repellent and insecticidal activities of *Hypericum* EO. 

The Bulgarian *Hypericum* species have been studied mainly for the content of hypericin, pseudohypericin, and flavonol glycosides (e.g., isoquercitrin and hyperoside) [23,24,25,26]. There is only one article on the EO of Bulgarian *H. maculatum* and *H. perforatum* [27]. However, there are no reports on the EO profile of Bulgarian *H. hirsutum*, *H. montbretii*, *H. cerastoides*, *H. rumeliacum*, and *H. calycinum*. There is very little data on the repellent, insecticidal, and antifungal activities of *Hypericum* EO. Therefore, this study aimed to (1) determine the EO composition of seven Bulgarian *Hypericum* species, and (2) evaluate the feasibility of *H. perforatum* EO as a potential biopesticide.

**Table 1:** Our data and literature data of phytochemical research on *H. perforatum* (HP); *H. cerastoides* (Hcer); *H. rumeliacum* (HR); *H. hirsutum* (Hh); *H. maculatum* (Hmac); *H. montbretii* (Hmon); and *H. calycinum* (Hcal).

Reference	Species	Distillation Type	Main Compounds in %	Country
Our data	HP1 ^1^	HD/hexane	*α*-pinene (6.76); *β*-pinene (3.04); *trans*-*β*-ocimene (5.28); *β*-caryophyllene (16.08); germacrene D (12.87); *γ*-cadinene (3.72); *δ*-cadinene (3.51); *trans*-nerolidol (2.25); spathulenol (3.95); caryophyllene oxide (5.12)	Bulgaria
	HP2	HD/hexane	*α*-pinene (6.2); *β*-pinene (3.5); *β*-myrcene (2.99); *trans*-*β*-ocimene (5.81); *β*-caryophyllene (6.19); germacrene D (16.8); *γ*-cadinene (3.13); *δ*-cadinene (3.54); spathulenol (3.5); caryophyllene oxide (3.35); *n*-hexadecanoic acid (4.95)	
	HP3	HD	2-methyloctane (10.33); *α*-pinene (10.61); 3-methylnonane (2.98); *β*-pinene (5.95); *trans-β*-caryophyllene (16.02); germacrene D (5.44); caryophyllene oxide (15.90)	
	Hmon	HD	*α*-pinene (15.2); *β*-pinene (5.14); *β*-myrcene (3.05); *cis-β*-ocimene (5.73); *trans-β*-ocimene (3.33); *β*-caryophyllene (4.2); *γ*-cadinene (5.75); *δ*-cadinene (4.63); *trans*-nerolidol (4.67); *n*-hexadecanoic acid (4.09); *n*-undecane (4.6)	
	Hcer	HD/hexane	ethyl 2-methyl pentanoate (6.81); thymol (8.31); thymol acetate (4.77); thymohydroquinone (3.63); *n*-hexadecanoic acid (36.48); 3,7,11,15-tetramethyl-2-hexadecen-1-ol (28.49); (*Z*,*Z*)-9,12-octadecadienoic acid (3.45); (*Z,Z,Z*)-9,12, 15-octadecatrienoic acid (4.03)	
	HR	HD/hexane	ethyl 2-methyl pentanoate (3.39); *α*-pinene (9.89); *β*-pinene (16.43); *β*-myrcene (5.95); *α*-phellandrene (2.93); D-limonene (3.75); *cis-β*-ocimene (8.69); *trans-β*-ocimene (12.88); *γ*-terpinene (3.32); dodecanal (2.53); germacrene D (3.82); *n*-nonacosane (2.45)	
	Hcal1	HD/hexane	*n*-nonane (5.33); *α*-pinene (23.86); *β*-pinene (29.37); *β*-myrcene (6.48); D-limonene (9.74); germacrene D (6.5)	
	Hcal2	HD/hexane	*α*-pinene (7.99); *β*-pinene (20.62); D-limonene (14.44); *cis-β*-ocimene (3.92); *β*-caryophyllene (4.15); *α*-humulene (6.70); germacrene D (8.84); *α*-muurolene (3.13); *α*-muurolol (torreyol) (5.66)	
	HP5	SD	2-methyloctane (9.23); *α*-pinene (8.77); *β*-pinene (3.08); neryl acetate (9.2); italicene (2.82); *β*-himachalene (7.58)	
	HP4	SD	2-methyloctane (40.89); nonane (8.80); α-pinene (13.75); 3-methylnonane (11.34); *β*-pinene (2.28); 2-methyldecane (5.29); undecane (6.24); caryophyllene oxide (1.97)	
	Hh	HD	nonane (47.80); undecane (18.54); *α*-longipinene (2.49); *trans-β*-farnesene (1.86); *α*-himachalene (2.04); caryophyllene oxide (3.05); cedrol (5.01)	
	Hmac	HD	2-methyloctane (2.29); nonane (8.64); *α*-pinene (6.63); 3-methylnonane (1.65); undecane (2.19); *β*-caryophyllene (3.85); *trans*-*β*-farnesene (4.15); *γ*-muurolene (2.36); germacrene D (26.77); *γ*-cadinene (2.07); *δ*-cadinene (5.88); caryophyllene oxide (2.33); ledol (3.62); *α*-epi-cadinol (2.67)	
[27]	Hmac	HD	*β*-caryophyllene (9.0); 3,7-dimethyl-1,3,6-octatriene (Z) (ocimene) (8.2)	Bulgaria
	HP		epi-bicyclosesquiphellandrene (10); *n*-alkanes	
[28]	HP	HD	caryophyllene oxide; *β*-caryophyllene; spathulenol; 1-tetradecanol; *β*-funebrene; 1-dodecanol; *γ*-muurolene	France
[17]	HP	SD	2-methyloctane (20.5); *n*-nonane (1.6); *α*-pinene (13.7); *β*-pinene (3.5); spathulenol (9.8); caryophyllene oxide (2.9); *n*-hexadecanoic acid (4.0)	Serbia, Montenegro
	HR	SD	*α*-pinene (0.8); *β*-pinene (1.5); *n*-undecane (0.9); germacrene D (5.8); *γ*-cadinene (4.8); spathulenol (1.9); caryophyllene oxide (2.4); *n*-dodecanoic acid (8.0); *n*-tetradecanoic acid (7.3); *n*-hexadecanoic acid (11.7)	
	Hmac	SD	*n*-nonane (14.9); *n*-undecane (5.9); spathulenol (7.0); globulol (8.0); *α*-cadinol (3.0); *n*-hexadecanoic acid (9.2); (*Z,Z,Z*)-9,12,15-octadecatrienoic acid, methyl ester (3.5)	
	Hh	SD	*n*-nonane (40.5); *n*-undecane (11.8); allo-aromadrene (3.8); *n*-hexadecanoic acid (3.1)	
[15]	HP	HD	*α*-pinene (6.5); *β*-pinene (3.6); E-*β*-ocimene (4.6); *E*-caryophyllene (11.2); germacrene D (18.6); bicyclogermacrene (5.0); 2-methyloctane (9.5)	Serbia
[19]	Hcal	microdistillation	*α*-pinene (6.6); *β*-pinene (29.2); limonene (7.2); *β*-caryophyllene (3.2); α-humulene (7.0); *α*-terpineol (11.5); *γ*-cadinene (4.7); humulene epoxide-II (3.7); spathulenol (4.4)	Kew Botanical Garden
[29]	HP	HD	caryophyllene oxide (7.7–29.5, flowers, 9.3–25.9, leaves); spathulenol (4.5–11.0 flowers, 6.4–15.7 leaves); viridiflorol (1.3–11.1, flowers, 0.5–9.5, leaves)	Lithuania
[7]	HP	SD	*α*-pinene (8.6); germacrene D (6.8); spathulenol (5.4); tetradecanol (3.8)	Serbia
	Hh	SD	nonane (24.8); undecane (13.3); 4-undecanone (4.1); *E*-caryophyllene (5.4)	
	HR	SD	*α*-pinene (18.5); *β*-pinene (21.5); myrcene (4.7); *p*-cymene (8.9); limonene (7.1); dodecanal (5.8)	
	Hmac	SD	nonane (5.5); *α*-pinene (4.4); undecane (3.5); spathulenol (6.8); globulol (10.2); T-muurolol (3.7)	
[30]	Hh	HD	*n*-undecane (32.2); patchoulene (11.8); caryophyllene oxide (9.3); *α*-longipinene (2.9); germacrene D (2.9); *α*-selinene (3.3); *n*-tetracosane (2.3)	Serbia
[31]	HP	HD	*α*-pinene (21.0); 2-methyl-octane (12.6); *β*-pinene (4.8); (*E*)-caryophyllene (5.8); *γ*-muurolene (6.9); caryophyllene oxide (6.4)	Greece
[32]	HP	HD	germacrene D (13.7%); *α*-pinene (5.1%); (*E*)-caryophyllene (4.7%); *n*-dodecanol (4.5%); caryophyllene oxide (4.2%); bicyclogermacrene (3.8%); spathulenol (3.4%)	Tajikistan
[13]	HP	HD	*β*-caryophyllene (11.7%); caryophellene oxide (6.3%); spathulenol (6.0%); *α*-pinene (5.0%)	Uzbekistan
[33]	HR	HD	*α*-pinene (43.80), *β*-pinene (9.82); dehydro-aromadendrene (6.81); *α*-copaene (5.41)	Greece
[34]	HP	HD	*α*-pinene (13.1); allo-aromadendrene (11.4); germacrene-D (10.6%); *n*-octane (7.3); *α*-selinene (6.5); *β*-selinene (5.5)	Tunisia
[35]	HP	HD	2-methyl-octane (12.3); *α*-pinene (20.9); *β*-pinene (4.7)	Greece
	HR	HD	*α*-pinene (40.3); *β*-pinene (9.7); *α*-copaene (5.3); dehydro-aromadendrene (6.6)	
[20]	HP	SDE	2-methyloctane (0.8–11.3); *n*-nonane (0.3–7.0); *α*-pinene (3.1–14.3); *β*-pinene (1.2–6.8); germacrene D (1.8–19.2); *γ*-cadinene (2.0–13.7); (-)-spathulenol (2.9–4.7); caryophyllene oxide (2.5–4.1); globulol (0.5–5.5); cadinol (0.9–5.0)	Estonia
[36]	HP	HD	*α*-pinene (1.5–36.74); *β*-pinene (0.30–6.89); spathulenol (0.0–6.05); caryophyllene oxide (1.15–12.35)	Albania
[37]	HP	HD	2,6-dimethyl-heptane (6.25–36.07%); *α*-pinene (5.56–26.03%); *β*-cadinene (0.0–22.58%); *γ*-cadinene (0.0–16.9%)	Iran
[38]	HP; Hcal; Hh	ethanol	quercetin-3-*O*-glucoside; quercetin-3-ogalactoside; quercitrin; quercetin; biapigenin	Sicily
[39]	HP	ethanol	naphthodianthrones (hypericin and its biosynthetic precursors); phloroglucinols (hyperforin and adhyperforin)	Italy
[40]	HP	HD	*α*-pinene (16.58); *β*-pinene (3.67); (*E*)-caryophyllene (9.52); *n*-nonane (13.59)	Croatia

^1^ HP1—*H. perforatum* location 1 (Odrinci); HP2—*H. perforatum* location 2 (Svirachi); HP3—*H. perforatum* location 3 (Uzana); HP4—*H. perforatum* (Com, USA); HP5—*H. perforatum* (Com, Bulgaria); Hcer—*H. cerastoides*; HR—*H. rumeliacum*; Hcal 1—*H. calycinum*, flos; Hcal 2—*H. calycinum*, leaves; Hh—*H. hirsutum*; Hmac—*H. maculatum*; Hmon—*H. montbretii*.; HD—hydrodistillation; SD—steam distillation; SDE—micro-simultaneous distillation–extraction.

## 2. Results and Discussion

As described in the Materials and Methods section, two independent experiments were performed in this study. The results for the EO composition of the studied *Hypericum* species were obtained in two different years, with different methodological setups, which is the reason why we do not compare them.

### 2.1. Essential Oil (EO) Compositions for the First Experiment

The EOs of *H. perforatum*, *H. maculatum*, and *H. hirsutum* by ClevA were extracted and analyzed in the first experiment. Analysis of variance (ANOVA) revealed a significant effect of species on the concentration of constituents, and further multiple means comparison results that show which of the species have statistically significantly different mean concentrations are presented in Table 2 and Table 3. The EO of *H. perforatum* by ClevA was compared with the EO of *H. perforatum* obtained via commercial (Com) steam distillation from Bulgaria (Com, Bul) and USA (Com, USA).

#### 2.1.1. Essential Oil (EO) Compositions of *H. perforatum*, *H. maculatum*, and *H. hirsutum* by ClevA

The compositions of the EOs isolated from *Hypericum* species of the first experiment are presented in Table 2 and Table 3, and Supplementary Tables S1–S3. Gas chromatography (GC) analyses identified between 26 and 50 constituents of *H. perforatum, H. maculatum, H. hirsutum*, and *H. montbretii* EOs (Supplementary Tables S1–S4). Overall, the EO constituents belonged to three classes, namely alkanes, monoterpenes, and sesquiterpenes (Table 2). This study found significant variability in the EO composition of the targeted *Hypericum* species. The main class of compounds of species from the section *Hypericum* (*H. maculatum, H. perforatum*) was the sesquiterpenes, with 66.37% for *H. maculatum* and 48.08% for *H. perforatum*, respectively. According to our results and previous research [7,28,37], the sesquiterpenes class was the predominant one for the section *Hypericum*. However, the results reported for eleven Albanian populations of *H. perforatum* were different from our results [36]. The latter authors found predominantly sesquiterpenes in six of the populations and predominantly monoterpenes in the other five populations of *H. perforatum* [36]. Apparently, there was great variation in *H. perforatum* EO composition and consequently, in the major classes of compounds [7,16,30,31,36,40]. 

Among chemicals, 2-methyloctane (10.88%), caryophyllene oxide (15.99%), *α*-pinene (10.61%), followed by germacrene D (5.44%), and *β*-pinene (5.95%) were the dominant compounds in the Bulgarian samples of *H. perforatum* EO (Table 2 and Table 3). Similar results but with different concentrations have been previously reported for *H. perforatum* EO from France [28], Serbia [7,40], and Greece [31,33] (Table 1). *γ*-Muurolene and carvacrol have been reported as the main compounds of *H. perforatum* EOs from Turkey [41] and Greece [26], while *epi*-bicyclosesquiphellandrene and *n*-alkanes were the main constituents of EOs of Bulgarian samples in a previous study [27]. These compounds (*γ*-muurolene, carvacrol, epi-bicyclosesquiphellandrene, and *n*-alkanes) were not detected in our study. Obviously, there is considerable variation in the composition of *H. perforatum* EO, which has been reported in other publications for different countries of origin [27,40,42] (Table 1). Based on the prevalent compounds of the EO, several chemical types of *H. perforatum* EO have been reported: *α*-pinene-type and caryophyllene-type from Albania [36] and *β*-caryophyllene-type, caryophyllene oxide-type, and germacrene D-type from Lithuania [29]. The EO of *H. perforatum* from this study (the first experiment) is a new chemical type of the species, containing 2-methyloctane, caryophyllene oxide, *α*-pinene, germacrene D, and *β*-pinene, respectively (Table 2 and Table 3). The composition of *H. perforatum* EO has been shown to be influenced by a number of factors such as the season, the location, the phenological stage as well as hybridization [18,21]. Due to its easy hybridization, there are many hybrids of *H. perforatum* with diploid, pentaploid, and triploid forms [9], which may also contribute to the observed diversity in its EO composition.

*Hypericum maculatum* belongs to the section *Hypericum*. Most of the previous research efforts on *H. maculatum* have focused on its flavonoid content, such as hyperoside, isoquercitrin, quercitrin, quercetin, and benzophenones [43,44]. Sesquiterpenes (66.37%) was the most abundant class of *H. maculatum* EO constituents in this study. This is contradictory to the findings published by Smelcerović et al. [17] that determined similarities in the contents of non-terpenes and sesquiterpenes between the EO profiles of species that belong to the section *Hypericum*. In the present study, the most abundant constituents of *H. maculatum* EO were germacrene D (26.77%), *δ*-cadinene (5.88%), nonane (8.66%), and *α*-pinene (6.63%) (ST3). In other studies, from Serbia and Bulgaria, *β*-caryophyllene, *δ*-cadinene, *γ*-muurolene, spathulenol, ocimene, nonane, (*E*)-*β*-farnesene, and globulol were found to be the most prevailing compounds in *H. maculatum* EO [7,17,27,40,45]. However, these compounds were found in very small quantities in the present study (Supplementary Table S3). 

The alkanes (67.19%) were the main class of *H. hirsutum* (section *Taeniocarpium*) EO constituents (Table 2, Supplementary Table S2). The main compounds in *H. hirsutum* EO in this study were nonane (48.19%), undecane (18.54%), and cedrol (5.04%) (Table 3, Supplementary Table S2). These results are in agreement with previous reports on *H. hirsutum* EO composition, also reporting high amounts of alkanes [7,17,30]. High amounts of *α*-pinene (88.3%) and (*E*)-caryophyllene (65.87%) of *H. hirsutum* EO were reported for samples from Turkey and Greece [46,47], while *cis*-guaiene was the prevailing EO constituent from Italy [46]. However, *α*-pinene, (*E*)-caryophyllene, and (*Z*)-guaiene were not identified in the present study. This study identified cedrol in the *H. hirsutum* EO and to our knowledge; it is the first report on cedrol in *Hypericum* EO. 

Generally, the chemical profiles of *H. hirsutum, H. montbretii*, and *H. perforatum* in this study were quite different, which contradicts the conclusion of Smelcerović et al. [17]. The cited authors found similarities in the contents of non-terpenes and sesquiterpenes in the EO between the sections *Hypericum* and *Taeniocarpium* [17].

Overall, the present study revealed that *H. perforatum*, *H. maculatum*, and *H. hirsutum*, EOs were significantly different with respect to the EO compositions. Secondary metabolites in plants have been successfully used as biomarkers in taxons [14,48]. There have been several published reports in which the authors looked for relationships between EO components as chemotaxonomic markers in the genus *Hypericum* [17,37,45,49]. However, because genetic and environmental conditions were the main factors that determined the composition of *Hypericum* EO, its components were deemed insufficient chemotaxonomical markers [17,48]. 

#### 2.1.2. The Comparison of *H. perforatum* EO by ClevA and the EO of *H. perforatum* Obtained via Commercial (Com) Steam Distillation

*Hypericum perforatum* has been traditionally used in herbal medicines for the treatment of various human disorders such as mild to moderate depression, wound healing, headaches, burns, puncture wounds, vertigo, and others. Generally, *Hypericum* spp. have low EO yield [42]; however, the EO of the *Hypericum* species has high value and is much sought after because of its strong antioxidant and antimicrobial activity [50]. Various EO extraction methods such as hydrodistillation, solvent extraction, and critical fluid extraction, have been used; however, steam distillation has been by far the most frequently used method by commercial producers [51]. According to literature data [51], chemical analysis of EOs obtained by different distillation methods revealed roughly the same compounds but in widely different percentages [51]. In this study, the EOs of *H. perforatum* (Com) from the USA and Bulgaria were compared with the EO of *H. perforatum* (Clev). The composition of EOs (Com) from the USA and Bulgaria were different and the results are presented in Table 2 and Table 3, and Supplementary Table S5. The alkanes were the prevailing chemical group of the EO from the USA (73.41%), while the EO from Bulgaria had monoterpenes and sesquiterpenes as the major group, at similar concentrations of 37.70% and 37.59%, respectively. This result is quite different from the EO profile obtained by the Clevenger apparatus. As mentioned above, 2-methyloctane (10.88%), caryophyllene oxide (15.99%), *α*-pinene (10.61%), followed by germacrene D (5.44%) and *β*-pinene (5.95%) were the predominant compounds in the EO obtained by hydrodistillation (Clev) in *H. perforatum.* 2-Methyloctane (40.89%), nonane (8.80%), *α*-pinene (13.75%), and 3-methylnonane (11.34%) were the main compounds in the commercial EO from USA (Com), while neryl acetate (9.2%), 2-methyloctane (9.13%), and *α*-pinene (8.70%) were the main compounds of the commercial EO from Bulgaria (Com) (Supplementary Table S5). The differences may be due to several factors, namely genetic and environmental conditions, and the very similar morphological characteristics of *Hypericum* species that might play a role in the identification and collection of some other *Hypericum* species alongside the *H. perforatum*. Due to the easy hybridization, *H. perforatum* is known to form hybrids with several other *Hypericum* species, and these hybrids have many transitional morphological forms [9,52]. Because of the transient forms of *H. perforatum*, herb pickers may not always precisely distinguish and collect *H. perforatum*. This may be a possible explanation for the observed differences in EO composition between the USA and Bulgaria (Com).

### 2.2. Essential Oil (EO) Compositions for the Second Experiment

In the second experiment, the EOs of *H. perforatum*, *H. cerastoides*, *H. rumeliacum*, *H. montbretii*, and *H. calycinum* (flower and leaves), extracted via hydrodistillation for 2 h 30 min in a modified Clevenger-type glass apparatus and collected with *n*-hexane were analyzed (Table 4 and Supplementary Table S6). *Hypericum montbretii* belongs to the section *Drosocarpium*, and the monoterpenes (38.09%) and sesquiterpenes (37.09%) were found in similar concentrations in the EO of this study (Table 2, Supplementary Table S4). Fifty-one components of the EO were detected in our study (Supplementary Table S4). *α*-Pinene (15.2), *cis-β*-ocimene (5.73), *β*-pinene (5.14), and *γ*-cadinene (5.75) components were the prevailing compounds of *H. montbretii* EO above 5.0% (Table 4). Other EO constituents included *β*-caryophyllene (4.2%), *δ*-cadinene (4.63%), (*E*)-nerolidol (4.67%), *n*-hexadecanoic acid (4.09%), and *n*-undecane (4.6%), all around the 4.0% level. We found only one article, by Erken et al. [41], on *H. montbretii* EO. The cited authors investigated a herbarium specimen of *H. montbretii* from Turkey and reported *α*-pinene (26%), *β*-pinene (19%), and undecane (5%) as the major compounds in the EO of that herbarium sample [41]. The EO of *H. montbretii* from Bulgaria was investigated for the first time in this study. 

The *H. cerastoides* EO had the most unique and dissimilar composition compared to that of the other *Hypericum* species. Ethyl 2-methyl pentanoate (6.87%), thymol (8.31%), hexadecanoic acid (36.48%), and 3,7,11,15-tetramethyl-2-hexadecen-1-ol (28.49%) were the prevailing compounds of its EO. The presence of fatty acids in the EOs of some species of the genus *Hypericum* has been previously reported for *H. tomentosum* L. [34], *H. perfoliatum* [35,53], *H*. *barbatum* Jacq., *H. rumeliacum*, *H. richeri* Vill.*, H. olympicum* L., and *H. adenotrichum* Spach. [17]. *Hypericum cerastoides* Spach EO was reported to contain *α*-pinene (58%), undecane (5%), and *β*-pinene (3%) based on herbarium specimens from Turkey [41]. Generally, the data on the *H. cerastoides* EO composition are scarce, and for Bulgaria, this is the first report.

*Hypericum rumeliacum*, section Drosocarpium, is a Balkan endemic species, found in Albania, Serbia, Greece, and Bulgaria. The EO of *H. rumeliacum* from different regions of Serbia and Greece has been extensively studied [7,17,35,49,54]. Bulgarian samples of this species have not been analyzed and reported previously. In this study, monoterpenes (72.06%) were found to be the main class of compounds with *α*-pinene (9.89%), *β*-pinene (16.43%), *β*-myrcene (5.95%), and *trans*-*β*-ocimene (12.88%) being the most abundant compounds in this group (Supplementary Table S6). When comparing the results of *H. rumeliacum* EO in this study with results obtained for the same species in previous reports [7,17,35,49,54], it appears that each of these studies found different dominant compounds. For example, 2-methyloctane (20.5%), *α*-pinene (13.7%), and spathulenol (9.8%) were the main EO compounds of samples from Serbia [17]. In another study from Serbia, Saroglou et al. [7] reported *α*-pinene (18.4%), *β*-pinene (21.5%), *p*-cymene (8.9%), D-limonene (7.1%), and dodecanal (5.8) to be the prevailing compounds. A large variation (also from Serbia) in the predominant constituents of the EO was reported by Djordjevic et al. [54] where the main components were *trans*-*β*-ocimene (18.2%), *β*-pinene (14.7%), *cis*-*β*-ocimene (13.0%), dodecanal (7.4%) germacrene D (5.8%), and myrcene (5.8%). However, an entirely different EO composition of samples also from Serbia was reported by Radulović and Blagojević [49]. The cited authors determined the EO composition in the flowering phase and in the fruit-forming phase [49]. They concluded that undecane (6.6%), dodecanal (10.8%), and germacrene D (14.1%) were prevailing at the flowering stage, and *α*-pinene (7.3%), *β*-pinene (26.1%), *cis*-*β*-ocimene (8.5%), *trans*-*β*-ocimene (10.2%), bicyclogermacrene (7.7%), and germacrene D (15.1%) were dominant in the fruit-forming phase [49]. Previous studies from Greece have shown that the major constituents of the EO were *α*-pinene (43.80%), *β*-pinene (9.82%), dehydro-aromadendrene (6.81%), and *α*-copaene (5.41%) [33]. After comparing previously published data on *H. rumeliacum* [7,17,35,49,54], and comparing them to our data, it seems that *α*-pinene and *β*-pinene are the most frequent main components of the *H. rumeliacum* EO. Generally, the EO composition of the *H. rumeliacum* was highly variable, and, apparently, it depends on many factors such as phenological stages, and ecological and genetic conditions.

*Hypericum calycinum* is a Tertiary relict species native to Southeastern Bulgaria, present in Strandja Nature Park as undergrowth in thermophilic oak forests [55]. According to the Red Book of Bulgaria [55] and The Bulgarian Biodiversity Act [56], the species is endangered (EN B1ab(i,ii) + 2ab(i,ii); C2a(ii)) and protected [55,56]. The species was locally naturalized in Eastern Europe and Eastern Asia [1] and it is widespread and cultivated as an ornamental plant, including in North America. Phytochemical investigations of *H. calycinum* have been focused mainly on flavonoids, flavonoid glycosides, hyperforin, and cyclohexadienone derivatives [57,58,59]. Previous research on EO content in this species is scarce. Phytochemical investigation of *H. calycinum* EO in this study showed slight differences in EO composition from flowers and leaves, and the differences were mostly quantitative (Table 4). Generally, the predominant class of the EOs was the monoterpenes, with 73.65% in the flower EO and 54.66% in the leaf EO. *β*-Pinene, *α*-pinene, D-limonene, and germacrene D were the prevailing compounds in flowers and leaves, and among them, *β*-pinene was the most abundant (Table 4, Supplementary Table S6). It can also be noted that in the EO of flowers, *n*-nonane (5.33%) and *β*-myrcene (6.48%) were in greater quantity than in the EO of leaves, while *α*-humulene (6.70%), *α*-muurolol (torreyol) (5.66%), *α*-muurolene (3.13%), and *β*-caryophyllene (4.15%) were characteristic of leaf EO (Supplementary Table S6). There are two literature reports on *H. calycinum* EO [19,41]. The main components of the EO of the herbarium specimen of this species were *α*-pinene (24%) and *β*-pinene (14%) [41], while *α*-pinene (6.6%), *β*-pinene (29.2%), limonene (7.2%), *β*-caryophyllene (3.2%), *α*-humulene (7.0%), and *α*-terpineol (11.5%) were the prevailing compounds in aerial shoots of *H. calycinum* [19].

### 2.3. The Pesticide Activity of Commercially (Com) Available H. perforatum EO from Bulgaria, Steam Distilled and Donated by Alta Oils, Bulgaria Oil (Com, Bul)

A commercially available EO of *H. perforatum* (Com), obtained via steam distillation from Bulgaria (Com, Bul) was used for testing the repellent, insecticidal, and antifungal activity in this study due to the very low yield of *H. perforatum* EO obtained by hydrodistillation (Clev). 

#### 2.3.1. Repellent and Insecticidal Activities of Commercial *H. perforatum* EO from Bulgaria (Com, Bul) against *Sitobion avenae* and *Rhopalosiphum padi*

The repellent and insecticidal activities of *H. perforatum* (Com, Bul) against *S. avenae* and *Rh. padi* are presented in Table 5 and Table 6. 

##### Insecticidal Activity of Commercial *H. perforatum* EO from Bulgaria (Com, Bul)

Aphids are economically important pests on agricultural crops and their control is difficult because (1) they reproduce particularly by parthenogenesis and by an amphisexual generation, and (2) they can easily develop resistance to insecticides. In conventional agriculture, the main method for aphid control is the use of chemical pesticides [60]. Since aphids easily develop resistance to chemical pesticides, it is sometimes necessary to increase the applied doses. However, excessive use and high doses of pesticides for aphid control are two of the reasons for the negative effects that pesticides have on human health and the environment [61]. The EOs are volatile compounds, and not only do they repel insects but also have contact and fumigant insecticidal activities, and they can affect insect pests through complex mechanisms [60,61]. Results from this study demonstrated that *H. perforatum* (Com, Bul) EO application at concentrations of 5%, 4.5%, and 3.5% had a strong insecticidal effect (100%) on both types of aphids within 24th h.

The application of *H. perforatum* EO at concentrations of 5% and 4.5% not only killed *S. avenae* and *Rh. padi* but also exhibited phytotoxicity on *Hordeum vulgare* leaves (Table 5). Regarding observed phytotoxicity, *H. perforatum* EO caused necrotic injuries on the leaves, as most EOs are phytotoxic [61]. At a concentration of 2.5%, the EO showed a lower efficacy for both types of aphids, 83% for *S. avenae* and 80.9% for *Rh. padi*, at the 24th h. With increasing duration of treatment (72th h), the insecticidal activity reached 100% (Table 5). Similar effectiveness of extracts from three *Hypericum* species (*H. heterophyllum*, *H. perforatum*, and *H. scabrum*) was reported by Yaman and Şimşek [62]. The cited authors found that the effectiveness of the extracts of the three *Hypericum* species was statistically significant depending on the duration of the exposure [62]. After 72th h exposure, mortality ranged from 4.3 to 94.1% for *Rhyzopertha dominica*, *Tribolium confusum*, and *Sitophilus oryzae*, respectively [62]. Moreover, these authors reported that leaf extracts of *H. perforatum* were more effective on *R. dominica*, while flower and stem extracts of *H. scabrum* showed a high toxicity effect on *T. confusum* and *S. oryzae* [62].

In general, the insecticidal activity of *H. perforatum* EO (Com, Bul) at concentrations of 1.5% and 1% was low, and it increased with increasing concentrations of the EO (Table 5). Our results are in agreement with the conclusions of Baȿ et al. [63] and Parchin and Ebadollahi [64], who found that, with increasing concentrations, *H. perforatum* EO increased the mortality of *Tenebrio molitor* L. (Coleoptera: Tenebrionidae) and *Tribolium castaneum* (Herbst) [63,64]. We should note that the compositions of *H. perforatum* found in the cited studies [62,63,64] were very dissimilar. For example, Parchin and Ebadollahi [64] reported the dominance of *n*-decane (59.58%), dodecane (12.93%), ethylcyclohexane (6.84%), 5-methylnonane (4.71%), 3-methylnonane (4.32%), and tetradecane (3.82%) in the *H. perforatum* EO obtained via steam distillation, while α-pinene (51.2%), 3-carene (7.3%), and *α*-caryophyllene (5.2%) were the main compounds of the EO in the study of Baȿ et al. [63] obtained via hydrodistillation. In our study, 2-methyloctane, *α*-pinene, *β*-himachalene, and neryl acetate were the predominant EO components of *H. perforatum* (Com, Bul) (Supplementary Table S5). Apparently, the interaction between the components in the EO of *H. perforatum* exhibits a strong insecticidal effect. Since *Hypericum* EO exhibits a strong insecticidal effect against *Rh. padi* (Bird Cherry-oat aphid) and *S. avenae* (English grain aphid), this EO has the potential to replace harmful chemical insecticides for aphid control.

##### Repellent Activity of the Commercial EOs of *H. perforatum* from Bulgaria (Com, Bul)

The repellent activity of *H. perforatum* EO was evaluated at 6 concentrations: 0%, 1%, 2.5%, 3.5%, 4.5%, and 5% on *Rh. padi* (Bird Cherry-oat aphid) and on *S. avenae* (English grain aphid) (Table 6). The highest repellent activity was observed with the EO applications at 5% and 4.5% for both types of aphids (Table 6). Similar results were found for *Juniperus sabina* EO (Male, Female) and *J. communis* L., *J. oxycedrus* L., *J. pygmaea* C. Koch., and *J. sibirica* Burgsd, where a 4.5% concentration rate had a stronger repellant effect on the *S. avenae* aphids than on the *Rh. padi* aphids [65,66]. A literature review yielded no studies on the repellent effect of *H. perforatum* EO. The repellent activity of the EO from aniseed (*Pimpinella anisum* L.), peppermint (*Mentha piperita* L.), and lemongrass (*Cymbopogon flexuosus* (Nees ex Steud.) W. Watson) against *Rh. padi* has been reported by Pascual-Villalobos et al. [67], who found that some EO constituents were active: carvone increased mobility, whilst *cis*-jasmone repelled *Rh. padi* at a very low dose (0.02 μL/cm^2^ of the treated leaf) [67].

#### 2.3.2. Antifungal Activity of Commercial EOs of *H. perforatum* from Bulgaria (Com, Bul) on Fungal Plant Pathogens

As indicated in Section 2.3, we used a commercial EO of *H. perforatum* from Bulgaria for testing pesticide activity. The EO of *H. perforatum* did not show antifungal activity against tested pathogenic fungi *R. solani*, *Fusarium* sp., *B. cinerea*, and *Aspergillus* sp. A moderate inhibitory effect was observed for *Colletotrichum* sp.: 9.61% at 1 µL mL^−1^ and 11.56% at 2 µL mL^−1^ (Table 7 and Table 8). The antifungal activity of the *H. perforatum* EO on the mycelial growth of the tested pathogens was variable on different days after the treatment. On the third day of the experiment, we observed higher growth of fungal colonies of *Fusarium* sp. (104% at 1 µL mL^−1^ and 125.5% at 2 µL mL^−1^), *B. cinerea* (108.54% at 1 µL mL^−1^ and 122.25% at 2 µL mL^−1^), and *Colletotrichum* sp. (101.3% at 1 µL mL^−1^ and 102% at 2 µL mL^−1^) in treated variants compared to the control. The high inhibition of the mycelial growth of *Aspergillus* sp. by the EO was seen only on the third day and decreased during the experiment. The diameter of the fungal colonies, except *Colletotrichum* sp., became equal on the ninth day in all tested variants.

It is likely that the more substantial initial growth of *Fusarium* sp. and *B. cinerea* was due to some of the EO components of *H. perforatum* that have a stimulatory effect on these phytopathogens. It was previously reported that the *H. hyssopifolium* and *H. heterophyllum* EOs increased the growth of some fungal species [3]. The inhibitory effect of EOs on pathogenic fungi depends on their application rate and the duration of the inhibition period [68]. Nosrati et al. [69] reported that samples treated with 1 µL of spearmint (*Mentha spicata* L.) EO showed a slow decrease in the antifungal activity against *Fusarium oxysporum f.* sp. *radicis-cucumerinum* throughout the incubation period.

Both *β*-caryophyllene oxide and *α*-terpineol were identified as constituents of the *Hypericum* species EO and have been previously reported as mycelial growth inhibitors against fungi [3]. High values of the inhibitory effect of the *Ocimum sanctum* L. EO against target filamentous fungi may be due to the characteristically high content of the monoterpenoid alcohol linalool and the phenylpropanoid estragole. It can be noted that eugenol, linalool, and thymol were among the plant constituents that have significant antifungal activity [70]. 

## 3. Materials and Methods

### 3.1. Plant Materials

Plant materials of *H. perforatum* L., *H. maculatum* Crantz., *H. hirsutum* L. were collected in 2019 for the first experiment (Table 9), while *H. cerastoides* (Spach) N. Robson, *H. rumeliacum*, Boiss., *H. montbretia* Spach., *H. calycinum* Mant. (flower) and *H. calycinum* (leaves), and *H. perforatum* were collected in 2020 for the second experiment (Table 9). The samples of *H. calycinum* were collected ex situ from the Experimental and Teaching Garden of the Agricultural University, Plovdiv, Bulgaria. Voucher specimens of all these species were deposited at the Herbarium of the Agricultural University, Plovdiv, Bulgaria (SOA).

### 3.2. Methods

#### 3.2.1. Essential Oil (EO) Extraction from the *Hypericum* Biomass Samples

Two separate experiments were conducted: (i) in the 2019 and (ii) 2020 cropping seasons. The two experiments were independent, so we did not compare the results between the different collection years.

##### First Experiment

In the first experiment, conducted in 2019, the plant materials of *H. perforatum*, *H. maculatum*, and *H. hirsutum* were collected in the flowering stage. The locations, coordinates, and altitude are shown in Table 9. The EOs were analyzed following extraction via hydrodistillation. The samples of the four species were dried in laboratory conditions, in a shady location. The 100 g samples (inflorescences with a small part of the stem) of each four *Hypericum* species were cut into small pieces and put in 2 L distillation Clevenger units (ClevA) (Laborbio Ltd. Sofia, Bulgaria, www.laborbio.com, accessed on Feb 8th, 2023). We used 800 mL of water, resulting in a 1:8 ratio of plant material to water. The EO extraction was performed via distillation for 2 h at the Research Institute for Roses and Medicinal Plants in Kazanluk, Bulgaria. Each extraction was performed in three replicates. The EO of *H. perforatum* (ClevA) was compared to the EO of commercially available *H. perforatum* from Bulgaria (Com, Bul) and from the USA (Com, USA), extracted via steam distillation. The commercial EO samples of *H. perforatum* were received from two companies: (1) purchased from Mountain Rose Herb, USA, Com, USA, (Eugene, OR, USA), and (2) donated by Alta Oils, Bulgaria, Com, Bul. (Kazanluk, Bulgaria).

##### Second Experiment

In the second experiment conducted in 2020, *H. cerastoides*, *H. rumeliacum*, *H. montbretii*, and *H. calycinum* (flower) and *H. calycinum* (leaves) samples were collected. The exact weight of the fresh materials of the target *Hypericum* species is shown in Table 9. The EOs of *Hypericum* species in the second experiment were extracted by hydrodistillation at the University of Food Technologies in Plovdiv. The EOs were extracted by hydrodistillation for 2 h 30 min in a modified Clevenger-type glass apparatus. Because of the low EO yield and difficulty with the oil collection, *n*-hexane was used to wash the sides of the apparatus and collect all the oil. Therefore, the EOs in the second experiment were dissolved in *n*-hexane.

#### 3.2.2. Gas Chromatography (GC)–Mass Spectrometry (MS) Analyses of the EOs

##### The First Experiment—Gas Chromatography–Mass Spectrometry–Flame Ionization Detection (GC–MS–FID) Essential Oil Analysis

GC–MS–FID analysis of *Hypericum hirsutum*, *Hypericum maculatum*, and *Hypericum perforatum* samples and *Hypericum perforatum* standard EOs from the first experiment was performed by placing 50 μL of oil (weight also recorded) into a 10 mL volumetric flask. Samples were brought to volume with chloroform. 

Oil samples were analyzed by GC–MS–FID on an Agilent (Santa Clara, CA, USA) 7890A GC system coupled to an Agilent 5975C inert XL MSD. Chemical standards and oils were analyzed using a DB-5 column (30 m × 0.25 mm fused silica capillary column, film thickness of 0.25 µm) operated using an injector temperature of 240 °C, column temperature of 60 to 240 °C at 3 °C/min and held at 240 °C for 5 min, helium as the carrier gas, an injection volume of 1 µL (split ratio 25:1), and an MS mass range from 50 to 600. The FID temperature was 300 °C. Post-column splitting was performed so that 50% of outlet flow proceeded to FID and 50% to mass spectrometry (MS) detection. 

Compounds were identified by Kovats Index analyses and comparison of mass spectra with those reported in the Adams and NIST mass spectra databases as well as a direct comparison of MS and retention time to authentic standards. Commercial standards of nonane, decane, 2-nonanone, undecane, decanal, *α*-longipinene, *trans*-caryophyllene, *trans*-*β*-farnesene, (+)-valencene, caryophyllene oxide, cedrol, sabinene, *β*-pinene, myrcene, *p*-cymene, nonanal, terpinen-4-ol, isoledene, *β*-caryophyllene, *α*-humulene, *trans*-*β*-farnesene, (+)-valencene, ledol, and 2-pentylfuran were obtained from Sigma-Aldrich (St. Louis, MO, USA). Germacrene D was obtained from Supelco (via Sigma-Aldrich, St. Louis, MO, USA). Compounds were quantified by performing area percentage calculations based on the total combined FID area. For example, the area for each reported peak was divided by the total integrated area from the FID chromatogram from all reported peaks and multiplied by 100 to arrive at a percentage. The percentage of a peak is a percentage relative to all other constituents integrated into the FID chromatogram.

##### Second Experiment GC–MS Analysis

The chemical composition of the investigated *Hypericum* essential oils from the second experiment in two repetitions was determined by GC–MS analysis. Compounds were separated using an Agilent 5890A gas chromatograph coupled with an Agilent 5795C MSD and fitted with a fused silica capillary column HP-5MS (5% phenylmethylpolysiloxane, 30 m × 0.25 mm i.d., 0.25 μm film thicknesses) (Agilent Technologies, Santa Clara, CA, USA). The temperature was programmed from 60 °C to 300 °C with 5 °C/min, held for 10 min; the injection volume was 1.0 μL and the split ratio was 50:1. The flow rate of the He (carrier gas) was 0.8 mL/min. Electron ionization (EI) mass spectra were recorded in the positive ion mode at 70 eV; acquisition mass range was 30–600 *m*/*z*. The ion source transfer and the line were set at 250 °C. 

GC analysis of the EO volatile components was performed using an Agilent 5890A gas chromatograph equipped with a flame ionization detector (FID) on an Agilent capillary column, HP-5 (30 m × 0.32 mm; film thickness 0.25 μm) (Agilent Technologies). The temperature program conditions were the same as with GC–MS analysis. The temperatures of the detector and the injector were 280 °C and 220 °C, respectively. FID temperature was set at 260 °C. The carrier gas was helium at a flow rate of 1.0 mL/ min^1^.

A mixture of aliphatic hydrocarbons from C6 to C32 (Sigma Aldrich, St. Louis, MO, USA) was injected into the GC system under the above temperature program in order to calculate the retention index (RI) of each compound in the samples and the percentage compositions of the individual components were obtained from electronic integration measurements using FID. 

Compound identification was carried out by comparing the retention time, RI, and mass spectra of the chromatographic peaks with those in the commercial NIST’08 (National Institute of Standards and Technology, Gaithersburg, MD, USA) and Adams libraries [71].

### 3.3. The Pesticide Activity of Commercial (Com) H. perforatum EO from Bulgaria (Bul) Obtained by Steam Distillation, Donated by Alta Oils, Bulgaria Oil (Com, Bul)

As mentioned in Section 2.3. the oil yield of *H. perforatum* EO obtained by hydrodistillation (Clev) was very low. Because of this, a commercial EO of *H. perforatum* from Bulgaria (Com, Bul), obtained by steam distillation, was used for testing repellent, insecticidal, and antifungal activity.

#### 3.3.1. Testing the Repellent and Insecticidal Activity of the EOs Obtained via Commercial (Com) Steam Distillation Donated by Alta Oils, Bulgaria

##### Colonization of *Rhopalosiphum padi* and *Sitobion avenae* for Insecticidal and Repellent Activity

Colonies of the *Rhopalosiphum padi* (Bird Cherry-oat aphid) and *Sitobion avenae* (English grain aphid) were maintained at the Entomology Laboratory of the Institute of Agriculture in Karnobat (42°38′54.51″ N, 27°21′60.56″ E), Bulgaria following the method described previously [65]. *Hordeum vulgare* Jess. subsp. *distichum* L., var. *erectum*, cv. Obzor was used for the two aphids. The barley plants were grown in containers under controlled conditions as follows: (1) a temperature of 23–24 °C, (2) 65% RH, and (3) a light:dark (L:D) cycle of 8:16 h. The aphids were introduced and infested the experimental plants when *H. vulgare* plants reached the 3rd leaf stage. 

##### The Insecticidal Activity of *H. perforatum* EO (Com, Bul) against *Rhopalosiphum padi* and *Sitobion avenae*

The insecticidal activity of *H. perforatum* EO (Com, Bul) was tested according to a method described in Konstantopoulou et al. [72]. The EO was applied at concentrations of 0% (control), 1%, 2.5%, 3.5%, 4.5%, and 5% in three replicates. Two species of adult wingless forms of aphids, *Rhopalosiphum padi* (Bird Cherry-oat aphid) and *Sitobion avenae* (English grain aphid), were used for insecticidal activity, in three replicates. The procedure of evaluating the insecticidal activity of the EO has been described previously [65]. *Hypericum perforatum* EO was diluted in an aqueous solution with an emulsifier of 0.1% polysorbate 80. The control (0%) was treated with a 0.1% aqueous solution of polysorbate 80. Two microliters of the solution (0%, 1%, 2.5%, 3.5%, 4.5%, and 5%) were applied directly to barley leaves with the aphid colonies. The leaves were then dried on a filter paper and transferred to Petri dishes as described by Konstantopoulou et al. [72]. The Petri dishes were covered with cheesecloth (44 g/m^2^). The effect of the application (knockdown or mortality) was observed after 24 and 72 h. The results (knockdown and mortality) were compared with controls. The efficacy of EO concentrations was calculated according to the Henderson–Tilton formula [73]:

(1) $$\left(1-\frac{T_{a}\times C_{b}}{T_{b}\times C_{a}}\right)\times 100\%$$

where T_a_—number insects after treatment; T_b_—number insects before treatment; C_b_—the number of insects in control before treatment plot; C_a_—the number of insects in control after treatment plot.

##### The Repellent Activity of *H. perforatum* EO (Com, Bul) against *Rhopalosiphum padi* and *Sitobion avenae*

The repellent activity of the EO of *H. perforatum* (Com, Bul) was tested at concentrations of 0%, 1%, 2.5%, 3.5%, 4.5%, and 5% in three replicates. Two microliters of EO was tested for repellency by using the Petri dish analysis according to Jiang et al. [74]. The *H. perforatum* EO (Com, Bul) was diluted with an aqueous solution with an emulsifier, 0.1% polysorbate 80, as described previously [65] with one treated leaf (with different concentration of EO), and one non-treated leaf (control, 0.1% polysorbate 80). The leaves were five cm long and positioned parallel at a distance of 2 cm between them, on a moistened filter paper in Petri dishes [74]. Ten leafless aphids were introduced into each Petri dish between the treated leaf and non-treated leaf (control). The Petri dishes were then covered with a cheesecloth (44 g/m^2^). The repellent effect was observed and recorded after 24 h. Descriptive statistical analyses of the data were performed by calculating the mean and the standard deviation (SD) values of the three replicates [75].

#### 3.3.2. Antifungal Activity of Commercial *H. perforatum* EO (Com) Obtained by Steam Distillation, Donated by Alta Oils, Bulgaria Oil (EO), on Fungal Plant Pathogens

Commercially available EO from *H. perforatum* was tested as a mycelial growth inhibitor against five important plant pathogenic fungal species. The tested pathogens *Rhizoctonia solani* (stem canker and black scurf), *Fusarium *sp. (fusarium dry rot), *Botrytis cinerea* (grey mold), *Colletotrichum *sp. (anthracnose of orange), and *Aspergillus *sp. (black mold) were stored at 4 °C in the culture collection in the Department of Phytopathology, at the Agricultural University in Plovdiv, Bulgaria. The fungal cultures were initially isolated from stored potato tubers (*Solanum tuberosum* L.), tomato fruits (*Lycopersicon esculentum* Mill), and orange fruits (*Citrus × sinensis*). The five strains were identified according to the characteristics of the fungus and Koch’s postulates.

An agar dilution method was used for the preliminary testing of the antifungal activity of *H. perforatum*, namely on five plant pathogens. The EO was diluted in potato dextrose agar (PDA) at two concentrations (1 µL mL^−1^ and 2 µL mL^−1^). PDA with EO was poured onto Petri dishes (90 mm/d). Discs (five mm) were cut from the periphery of a 10-day-old culture of tested fungi and aseptically put in the center of the Petri dishes. Pure PDA medium with sterile distilled water (without EO) was used as the control. The inoculated Petri dishes were placed at 22 °C for nine days. All experiments were conducted in four replications. The diameter of the fungal colony was measured on the 3rd, 6th, and 9th day. The percent inhibition of the radial growth of the tested fungi was calculated using the following formula:

(DC − DT)/DC × 100% (2)

where DC is the diameter of the control colony, and DT is the diameter of the treatment colony. Mean and SD values were also calculated.

### 3.4. Statistical Analyses

For the data obtained from the first experiment, analysis of variance (ANOVA) of a completely randomized design (CRD), also known as one-way ANOVA, was conducted to determine the effect of species (five levels: *H. hirsutum*, *H. maculatum, H. perforatum, H. perforatum* (Com, USA), and *H. perforatum* (Com, Bul), on the concentrations of 15 constituents (2-methyloctane, nonane, *α*-pinene, 3-methylnonane, *β*-pinene, (*Z*)-*β*-ocimene, (*E*)-*β*-ocimene, undecane, caryophyllene oxide, (*E*)-caryophyllene, (*E*)-*β*-farnesene, germacrene D, *δ*-cadinene, *α*-epi-cadinol, and *α*-cadinol), and four classes (monoterpenes, sesquiterpenes, alkanes, and other). The analyses were completed using the GLM Procedure of SAS [76]. Since the effect of species was significant (*p*-value < 0.05) on the concentrations of all 19 constituents, further multiple means comparison was completed using Tukey’s multiple range test at 5% level of significance and letter groupings were generated. For each response variable, the validity of the normal distribution of the error terms assumption was verified by generating a normal probability plot of residuals and testing for normality of the error terms using the residuals, and the validity of the constant variance assumption of the error terms was verified by plotting the residuals vs. the fitted values, as described in Montgomery [75].

For the data from the second experiment, descriptive statistics (mean and standard deviation) were calculated using the three replicates. 

## 4. Conclusions

Generally, the EOs of the seven *Hypericum* species from Bulgaria had very different compositions, especially *H. perforatum*. The testing of EOs of *H. hirsutum*, *H. montbretii*, *H. cerastoides*, *H. rumeliacum*, and *H. calycinum* in Bulgarian populations was conducted for the first time. The application of *H. perforatum* EO (Com) at concentrations of 5% and 4.5% exhibited high repellent activity and was effective against two aphid species: *S. avenae* and *Rh. padi.* In our study, *H. perforatum* EO did not exhibit substantial antifungal activity against *R. solani*, *Fusarium* sp., *B. cinerea*, and *Aspergillus* sp. but had a moderate inhibitory effect on *Colletotrichum* sp. Since there is great variability in the compositions of *Hypericum* EOs, it is necessary to select and grow a specific accession with a desirable composition in order to standardize the EO composition. The standardized EO compositions are considered alternative products with the potential to substitute synthetic pesticides in controlling pests on agricultural crops. These molecules constitute a significant source of biologically active components—antioxidant, antibacterial, insecticidal, fungicidal, and herbicidal. Therefore, EOs have potential as biological products in integrated and ecological plant protection. 

## Figures and Tables

**Table 2:** Mean concentrations (%) of *trans-β*-farnesene, germacrene D, *δ*-cadinene, *α*-epi-cadinol, *α*-cadinol, monoterpenes, sesquiterpenes, alkanes, and other compounds obtained from the six species.

Species	*Trans*-*β*-Farnesene	Germacrene D	*δ*-Cadinene	*α*-Epi-cadinol	*α*-Cadinol	Monoterpenes	Sesquiterpenes	Alkanes	Other
Hh	<0.01 d ^1^	<0.01 d	<0.01 e	<0.01 c	<0.01 b	<0.01 e	19.72 d	67.19 b	12.85 b
Hmac	4.15 a	26.77 a	5.88 a	2.67 a	3.43 a	8.82 d	66.37 a	13.18 c	11.90 b
HP3	1.84 b	5.44 b	1.51 b	<0.01 c	<0.01 b	19.33 b	48.08 b	14.23 c	19.36 a
HP4	<0.01 d	<0.01 d	0.44 d	<0.01 c	<0.01 b	16.55 c	6.41 e	73.41 a	1.97 c
HP5	0.56 c	0.54 c	0.83 c	0.21 b	<0.01 b	37.70 a	37.59 c	2.69 d	19.17 a

HP3—*H. perforatum* location 3 (Uzana); HP4—*H. perforatum* (Com, USA); HP5—*H. perforatum* (Com, Bul); Hh—*H. hirsutum*; Hmac—*H. maculatum*. ^1^ Within each column, means followed by the same letter are not significantly different at the 5% level of significance using Tukey’s multiple means comparison method.

**Table 3:** Mean concentrations (%) of 2-methyloctane, nonane, *α*-pinene, 3-methylnonane, *β*- pinene, *cis-β*-ocimene, *trans*-*β*-ocimene, undecane, caryophyllene oxide, and *β*-caryophyllene obtained from the six species.

Species	2-Methyloctane	Nonane	α-Pinene	3-Methylnonane	*β*-Pinene	Cis-*β*-Ocimene	*Trans*-*β*-Ocimene	Undecane	CaryoPhyllene Oxide	*β*-CaryoPhyllene
Hh	<0.01 e ^1^	48.19 a	<0.01 e	0.51 d	<0.01 e	<0.01 d	<0.01 c	18.54 a	3.05 b	<0.01 d
Hmac	2.29 d	8.66 b	6.63 d	1.65 c	0.72 d	0.26 c	<0.01 c	2.19 c	2.33 c	3.85 b
HP3	10.88 b	1.35 d	10.61 b	2.98 b	5.95 a	0.42 a	0.80 a	0.92 d	15.90 a	16.02 a
HP4	40.89 a	8.80 b	13.75 a	11.34 a	2.28 c	<0.01 d	<0.01 c	6.24 b	1.97 d	1.70 c
HP5	9.13 c	1.79 c	8.70 c	0.00 e	3.05 b	0.29 b	0.35 b	<0.01 e	0.30 e	<0.01 d

HP3—*H. perforatum* location 3 (Uzana); HP4—*H. perforatum* (Com, USA); HP5—*H. perforatum* (Com, Bul); Hh—*H. hirsutum*; Hmac—*H. maculatum*. ^1^ Within each column, means followed by the same letter are not significantly different at the 5% level of significance using Tukey’s multiple means comparison method.

**Table 4:** Mean concentrations (%) of *α*-pinene, *β*-pinene, *β*-myrcene, (*E*)-*β*-ocimene, *β*-caryophyllene, *β*-farnesene, germacrene D, caryophyllene oxide, monoterpenes, sesquiterpenes, long-chain alkane, and fatty acid of four *Hypericum* species extracted by *n*-hexane.

Compound Name	HP1 ^1^	HP2	HCer	HR	HCal, 1	HCal, 2	Hmon
*α*-Pinene	6.76	6.41	nd	9.89	23.86	7.99	15.26
*β*-Pinene	3.04	3.51	nd	16.43	29.37	20.62	5.06
*β*-Myrcene	2.52	3.00	nd	5.95	6.48	2.39	nd
(E*)-β*-Ocimene	5.28	5.81	nd	12.88	0.61	0.65	5.68
*β*-Caryophyllene	16.08	6.20	nd	0.79	1.53	4.15	4.09
*β*-Farnesene	4.05	6.03	nd	0.11	1.02	0.45	0.93
Germacrene D	12.87	16.08	nd	3.82	6.50	8.84	2.58
Caryophyllene oxide	5.12	3.35	nd	0.16	0.16	0.40	1.25
Monoterpenes	19.55	22.69	8.31	72.06	73.65	54.96	37.09
Sesquiterpenes	69.61	68.71	3.63	11.37	15.76	37.06	38.09
Long-chain alkane/alkane	2.66	1.31	2.59	4.28	1.68	1.19	4.94
Fatty acid	3.36	0.79	43.96	nd	nd	nd	nd

^1^ HP1—*H. perforatum* location 1 (Odrinci); HP2—*H. perforatum* location 2 (Svirachi); HCer—*H. cerastoides*; HR—*H. rumeliacum*; Hmon—*H. montbretii;* HCal 1—*H. calycinum*, flos; HCal 2—*H. calycinum*, leaves; nd—not detected.

**Table 5:** The insecticidal effect of commercial EOs of *H. perforatum* from Bulgaria (Com, Bul) on two aphids *(Rhopalosiphum padi* and *Sitobion avenae)* (mean ± SD).

EO Concentrations (%)	After 24 h in % ± SD		After 72 h in % ± SD	
	*S. avenae*	*Rh. padi*	*S. avenae*	*Rh. padi*
5	100 ± 0.00	100 ± 0.00	*	*
4.5	100 ± 0.00	100 ± 0.00	*	*
3.5	100.0 ± 0.00	100.0 ± 0.00	*	*
2.5	83.0 ± 1.15	80.90 ± 2.8	*	*
1.5	89.0 ± 1.00	89.08 ± 0.57	90.50 ± 1.15	90.62 ± 1.00
1	0.30 ± 3.60	49.97 ± 3.05	59.00 ± 3.78	51.93 ± 2.51

* The values are not shown because the aphids were died at 24 h.

**Table 6:** Means of the repellent activities obtained from the concentrations of commercial EO of *H. perforatum* from Bulgaria (Com, Bul) against *Rhopalosiphum padi* and *Sitobion avenae* (mean ± SD).

EO Concentrations (%)	% nb/leaf *S. avenae* ± SD		% nb/leaf *Rh. padi* ± SD	
	Treated Plant	Control	Treated Plant	Control
5	0.33 ± 0.57	4.00 ± 1.00	0.67 ± 0.57	1.67 ± 1.53
4.5	0.33 ± 0.57	6.00 ± 1.00	0.33 ± 0.57	7.00 ± 1.00
3.5	1.33 ± 0.58	6.00 ± 2.00	1.00 ± 0.00	6.00 ± 2.00
2.5	1.67 ± 0.58	7.67 ± 0.58	1.33 ± 1.15	8.00 ± 1.00
1.5	0.67 ± 1.15	8.67 ± 1.15	1.00 ± 1.00	8.33 ± 1.52
1	1.33 ± 0.58	8.33 ± 0.58	1.67 ± 0.58	8.33 ± 0.58

**Table 7:** Inhibitory effect of commercial *Hypericum perforatum* essential oils (Com, Bul) on plant pathogenic fungi (mean ± SD).

Pathogens/Day of Report	*Fusarium* sp.		*Botrytis cinerea*		*Rhizoctonia solani*	
	Control	*Hypericum* EO	Control	*Hypericum* EO	Control	*Hypericum* EO
Diameter of Radial Mycelial Growth (mm)						
1 µL mL^−1^						
3rd day	24.68 ± 0.54	25.65 ± 0.57 (104) ^1^	25.63 ± 0.77	27.82 ± 0.85 (108.5)	30.18 ± 1.09	28.53 ± 0.49
6th day	57.43 ± 0.60	58.63 ± 0.62 (102)	62.63 ± 2.46	62.50 ± 4.36	58.70 ± 0.48	57.70 ± 0.56
9th day	85.00 ± 0.00	85.00 ± 0.00	85.00 ± 0.00	85.00 ± 0.00	85.00 ± 0.00	85.00 ± 0.00
PI	0.00 ± 0.00		0.00 ± 0.00		0.00 ± 0.00	
2 µL mL^−1^						
3rd day	24.70 ± 1.57	31.00 ± 3.34 (125.5)	26.38 ± 1.11	32.25 ± 1.50 (122.3)	31.43 ± 0.43	30.25 ± 1.26
6th day	59.25 ± 1.09	59.63 ± 1.70	60.80 ± 2.93	61.88 ± 1.31	59.13 ± 0.89	58.38 ± 1.11
9th day	85.00 ± 0.00	85.00 ± 0.00	85.00 ± 0.00	85.00 ± 0.00	85.00 ± 0.00	85.00 ± 0.00
PI	0.00 ± 0.00		0.00 ± 0.00		0.00 ± 0.00	

^1^ Values in parentheses represent the percentage of the variant relative to control.

**Table 8:** Inhibitory effect of commercial *Hypericum perforatum* essential oils on plant pathogenic fungi (mean ± SD).

Pathogens/Day of Report	*Colletotrichum* sp.		*Aspergillus* sp.	
	Control	*Hypericum* EO	Control	*Hypericum* EO
Diameter of Radial Mycelial Growth (mm)				
1 µL mL^−1^				
3rd day	20.43 ± 0.43	20.70 ± 0.43	42.00 ± 0.36	28.20 ± 0.71
6th day	47.83 ± 0.72	48.00 ± 0.16	77.83 ± 0.77	73.88 ± 0.48
9th day	83.38 ± 1.82	75.33 ± 0.54	85.00 ± 0.00	85.00 ± 0.00
PI	9.61 ± 2.36		0.00 ± 0.00	
2 µL mL^−1^				
3rd day	22.85 ± 3.21	23.13 ± 1.31	42.70 ± 0.53	32.88 ± 0.85
6th day	56.25 ± 2.50	57.25 ± 1.50	77.58 ± 0.81	75.00 ± 0.41
9th day	81.78 ± 1.00	72.25 ± 1.26	85.00 ± 0.00	85.00 ± 0.00
PI	11.56 ± 1.64		0.00 ± 0.00	

**Table 9:** Location coordinates, altitude (Masl), and sample size (g) of analyzed *Hypericum* species from Bulgaria.

Species	Abbreviation	Habitat	Location	Masl	Distillation Type	Samples, g (Inflorescences)
**Section *Hypericum***						
*H. maculatum* Crantz.	Hmac	Uzana	42°45′48.8″ N 25°08′42.7″ E	1438	HD	100
*H. perforatum* L.	HP1	Odrinci	41°26′59.4″ N 26°07′53.5″ E	105	HD	77
	HP2	Svirachi	41°44′98.9″ N 26°13′53.8″ E	89	HD	125
	HP3	Uzana	42°45′48.8″ N 25°08′42.7″ E	1438	HD	100
	HP4, USA	commercial			SD	
	HP5, BUL	commercial			SD	
*H. maculatum* Crantz.	Hmac	Uzana	42°45′48.8″ N 25°08′42.7″ E	1438	HD	100
*H. perforatum* L.	HP1	Odrinci	41°26′59.4″ N 26°07′53.5″ E	105	HD	77
	HP2	Svirachi	41°44′98.9″ N 26°13′53.8″ E	89	HD	125
	HP3	Uzana	42°45′48.8″ N 25°08′42.7″ E	1438	HD	100
**Section *Campylopus* (Spach) Endl.**						
*H. cerastoides* (Spach) N. Robson	Hcer	above h. Zdravetc	42°00′36.1″ N 24°69′27.9″ E	1299	HD	58
**Section *Drosocarpium* Spach**						
*H. rumeliacum* Boiss.	HR	Novo selo	42°09′97.6″ N 24°46′75.2″ E	311	HD	125
*H. montbretii* Spach.	Hmon	Mandrica	41°43′45.5″ N 26°14′82.4″ E	89	HD	100
**Section *Eremanthe* (Spach) Endl.**						
*H. calycinum* Mant.	Hcal	ex situ	42°13′31.9″ N 24°76′61.1″ E	167	HD	78, flower 68, leaves
**Section *Taeniocarpium* Jaub. & Spach**						
*H. hirsutum* L.	Hh	Uzana	42°45′48.8″ N 25°08′42.7″ E	1438	HD	100

## Data Availability

Data of the compounds are available from the authors.

## Supplementary Materials

The following supporting information can be downloaded at: https://www.mdpi.com/article/10.3390/plants12040923/s1, Table S1: Essential oil composition of *Hypericum perforatum*; Table S2: Essential oil composition of *Hypericum hirsutum*; Table S3: Essential oil composition of *Hypericum maculatum*; Table S4: Essential oil composition of *Hypericum montbretii*; Table S5: Essential oil composition of *Hypericum perforatum*, commercial from USA and Bulgaria; Table S6: Essential oil composition of *Hypericum perforatum, H. rumeliacum* from the second experiment.

## Abbreviations

ST	supplementary table;
EO	essential oils;
GC-MS-FID	Gas Chromatography–Mass Spectroscopy–Flame Ionization Detection analyses;
ClevA	Clevenger apparatus;
Com	commercial;
HP1	*H. perforatum* location 1 (Odrinci);
HP2	*H. perforatum* location 2 (Svirachi);
HP3	*H. perforatum* location 3 (Uzana);
HP4	*H. perforatum* (Com, USA);
HP5	*H. perforatum* (Com, Bulgaria);
HCer	*H. cerastoides*;
HR	*H. rumeliacum*;
HCal 1	*H. calycinum*, flos;
HCal 2	*H. calycinum*, leaves;
Hh	*H. hirsutum*;
Hmac	*H. maculatum*;
Hmon	*H. montbretii*;
HD	hydrodistillation;
SD	steam distillation;
SDE	micro simultaneous distillation extraction;
Masl	meters above sea level.
