# Supplementary material for: Essential Oil Composition of Seven Bulgarian Hypericum Species and Its Potential as a Biopesticide
Source: Plants (Basel). 2023 Feb 17;12(4):923. doi: 10.3390/plants12040923 (PMC9962312; doi:10.3390/plants12040923)
Supplement: Supplementary file 1 [file plants-12-00923-s001.zip › plants-2217402-supplementary.pdf]

SUPPLEMENTARY TABLES

Table S1. Essential oil composition of *Hypericum perforatum*

Observed KI*	Adams KI	Compound Name	Min-Max (%)
-	865	2-methyloctane	10.79-10.96
-	900	nonane	1.34-1.36
921	924	α-thujene	0.27
929	932	α-pinene	10.52-10.72
962	972	3-methylnonane	2.95-3.03
971	974	β-pinene	5.90-6.03
983	984	myrcene	0.46-0.47
1019	1020	p-cymene	0.57-0.58
1023	1024	limonene	0.44-0.45
1031	1032	cis-β-ocimene	0.41-0.43
1042	1044	trans-β-ocimene	0.79-0.81
1059	1061	2-methyldecane	1.03-1.07
1099	1100	undecane	0.88-0.98
1129	-	unknown, mw 126	0.55-0.59
1173	1174	terpinen-4-ol	0.28-0.29
1186	1186	α-terpineol	0.29-0.30
1261	1261	2-methyldodecane	0.27-0.31
1371	1374	α-copaene	0.30-0.31
1379	1387	β-bourbonene	0.33-0.37
1406	1411	2-epi-β-funebrene	0.35-0.36
1415	1417	trans-β-caryophyllene	15.78-16.21
1440	-	unknown, 204	0.31-0.32
1450	1452	α-humulene	0.75-0.89
1453	1454	trans-β-farnesene	1.85-1.86
1457	1458	alloaromadendrene	0.34-0.49
1475	1478	γ-muurolene	1.36-1.39
1480	1480	germacrene D	5.37-5.47
1494	-	unknown, 204	1.33-1.36
1500	1500	α-muurolene	0.36-0.37
1512	1513	γ-cadinene	0.80-0.82
1521	1522	δ-cadinene	1.49-1.53
1548	-	unknown, 220	0.98-0.99
1557	-	unknown, 220	0.16-0.34
1563	-	unknown, 204	0.43-0.71
1573	1577	spathulenol	2.00-2.05
1577	1582	caryophyllene oxide	15.70-16.11
1595	1602	ledol	1.26-1.27
1599	-	unknown, 220	0.46-0.47
1627	-	unknown, 222	0.76-0.78
1630	-	unknown, 220	0.67-0.69
1636	-	unknown, 218	0.87-0.92
1645	-	unknown, 222	0.37-0.41
1650	-	unknown, 222	0.85-0.93
1654	-	unknown, 220	0.47-0.58
1671	-	unknown, 220	1.00-1.05
1760	-	unknown	0.58-0.72

1839	-	unknown	0.37-0.39
1957	-	unknown	0.76-0.99
2094	-	unknown	0.40-0.43
2108	-	unknown	0.69-0.77
2292	-	unknown	0.33-0.36
2494	-	unknown	0.42-0.52

*** Kovats Index**

Table S2. Essential oil composition of *Hypericum hirsutum*

Observed KI*	Adams KI	Compound Name	Min-Max (%)
-	900	nonane	47.04-48.3
962	972	3-methylnonane	0.51-0.52
993	1000	decane	0.84-0.85
1089	1087	2-nonanone	0.41-0.45
1101	1100	undecane	18.36-18.67
1199	1201	decanal	0.27-0.29
1348	1350	α-longipinene	2.46-2.53
1414	1417	trans-caryophyllene	1.49-1.55
1431	1432	α-trans-bergamotene	0.39-0.42
1448	-	unknown, 204	6.75-7.05
1454	1454	trans-β-farnesene	1.76-1.94
1475	1478	γ-muurolene	1.10-1.15
1482	1481	γ-himachalene	1.97-2.12
1492	1496	valencene	0.65-0.68
1500	-	unknown, 204	0.33-0.35
1513	1513	γ-cadinene	0.44-0.48
1522	1522	δ-cadinene	1.08-1.16
1561	-	unknown, 220	0.18-0.26
1572	-	unknown, 220	0.59-0.64
1576	1582	caryophyllene oxide	2.98-3.13
1594	1600	cedrol	4.87-5.21
1643	-	unknown, 204	0.49-0.50
1651	-	unknown, 222	0.57-0.61
1682	-	unknown, 204	0.21-0.24
1761	-	unknown, 330	0.33-0.38
1958	-	unknown	0.86-0.97
2078	-	unknown	0.44-0.49
2096	-	unknown	0.35-0.36
2293	-	unknown	0.38-0.39

*** Kovats Index**

Table S3. Essential oil composition of *Hypericum maculatum*

Observed KI*	Adams KI	Compound Name	Min-Max (%)
-	865	2-methyloctane	2.28-2.31
-	900	nonane	8.59-8.71
929	932	α-pinene	6.59-6.67
962	972	3-methylnonane	1.64-1.66
967	969	sabinene	0.47
971	974	β-pinene	0.71-0.73
984	988	myrcene	0.32-0.33
1019	1020	p-cymene	0.18
1024	1024	limonene	0.26
1043	1044	(E)-β-ocimene	0.26
1100	1100	undecane	2.17-2.22
1106	1100	nonanal	0.36-0.37
1173	1174	terpinen-4-ol	0.23
1347	1351	α-cubebene	0.35-0.36
1371	1374	α-copaene	0.87
1380	1384	β-bourbonene	0.99-1.00
1384	1390	β-cubebene	0.36-0.37
1403	1409	α-gurjunene	0.98-1.00
1414	1417	trans-caryophyllene	3.82-3.88
1424	1430	β-copaene	0.65-0.67
1441	-	unknown, 204	0.66-0.67
1450	1452	α-humulene	0.57-0.74
1455	1454	trans-β-farnesene	3.91-4.28
1458	1458	alloaromadendrene	0.97-1.72
1477	1478	γ-muurolene	2.32-2.42
1483	1480	germacrene D	26.61-27.0
1495	-	unknown, 204	1.15-1.18
1497	1500	bicyclogermacrene	1.55-1.60
1500	1500	α-muurolene	0.98-0.99
1513	1513	γ-cadinene	2.06-2.07
1522	1522	δ-cadinene	5.85-5.93
1535	1537	α-cadinene	0.41-0.42
1539	1544	α-calacorene	0.26
1572	1577	spathulenol	1.86-1.88
1576	1582	caryophyllene oxide	2.31-2.36
1595	1602	ledol	3.54-3.62
1637	1638	τ-cadinol	2.65-2.69
1642	1646	α-muurolol	0.77-0.78
1651	1652	α-cadinol	3.40-3.45
1761	-	unknown, 218	0.60-0.67
1896	1900	nonadecane	0.40-0.41
1958	-	unknown	0.84-0.90
2079	-	unknown	1.41-1.48
2095	-	unknown	0.40-0.42
2109	-	unknown	0.46-0.50
2296	-	unknown	0.32-0.33

*** Kovats Index**

Table S4. Essential oil composition of *Hypericum montbretii*

**Observed KI***	**Adams KI**	Name	Average (%)
901	900	n-Nonane	0.54-0.53
926	924	α-Thujene	0.07-0.09
935	932	α-Pinene	15.19-15.32
968	969	Sabinene	0.31-0.32
973	974	β-Pinene	5.14-4.98
985	988	β-Myrcene	3.05-3.37
1015	1014	α-Terpinene	0.15-0.16
1022	1020	p-Cymene	0.16-0.17
1025	1024	D-Limonene	2.6-2.62
1027	1026	β-Phellandrene	0.07-0.09
1034	1032	cis-β-Ocimene	5.64-5.73
1040	1044	trans-β-Ocimene	2.88-3.33
1055	1054	γ-Terpinene	0.54-0.56
1066	1063	n-Octanol	0.21-0.22
1085	1086	Terpinolene	0.40-0.43
1101	1100	n-Undecane	3.80-4.59
1103	1102	Nonanal	0.76-0.81
1178	1174	1-Terpinen-4-ol	0.39-0.42
1268	1271	trans-2-decenol	0.89-1.01
1347	1351	α-Cubebene	2.90-3.02
1373	1374	α-Copaene	0.28-0.37
1390	1388	β-Bourbonene	0.27-0.29
1403	1399	Dodecanal	0.84-0.87
1419	1417	β-Caryophyllene	3.99-4.20
1436	1439	Aromadendrene	0.41-0.43
1442	1440	β-Farnesene	0.90-0.95
1453	1343	α-Humullene	0.45-0.48
1484	1481	Germacrene D	2.51-2.64
1502	1500	α-Muurolene	1.73-1.82
1510	1513	γ-Cadinene	5.50-5.75
1521	1522	δ-Cadinene	4.40-4.63
1564	1561	trans-Nerolidol	4.67-5.03
1579	1577	(-)-Spathulenol	1.08-1.14
1580	1582	Caryophyllene oxide	1.22-1.29
1633	1630	γ-Eudesmol	1.96-2.26
1637	1638	tau.-Cadinol	0.89-0.98
1642	1640	tau.-Muurolol	0.49-0.51
1647	1649	α-Muurolol (Torreyol)	1.40-1.47
1650	1652	α-Eudesmol	1.60-1.69
1777	1779	α-14-hydroxy-Muurolene	3.16-3.40
1871	1874	1-Hexadecanol	0.42-0.43
1900	1900	n-Nonadecane	0.35-0.37
1955	1959	n-Hexadecanoic acid	4.86-4.96
2101	2100	n-Heneicosane	0.51-0.53
2122	2118	3,7,11,15-Tetramethyl-2-hexadecen-1-ol	0.23-0.24
2144	2141	(Z,Z,Z)-9,12, 15-octadecatrienoic acid	1.85-1.92
2302	2300	n-Tricosane	0.20-0.21
2501	2500	n-Pentacosane	0.25-0.26
2803	2800	n-Octacosane	0.94-0.98

2900		n-Nonacosane	2.59-2.70

*** Kovats Index**

Table S5. Essential oil composition of *Hypericum perforatum,* commercial from USA and Bulgaria

Observed KI*	Adams KI	Compound Name (from USA)	Min-Max (%)
-	865	2-methyloctane	40.86-41.00
-	900	nonane	8.80-8.82
929	932	α-pinene	13.73-13.75
942	946	camphene	0.13
963	972	3-methylnonane	11.30-11.37
971	975	β-pinene	2.27-2.28
983	1000	2-pentylfuran	0.13-0.19
993	1000	decane	0.24
1019	1020	p-cymene	0.59
1023	1024	limonene	0.76-0.77
1060	1061	2-methyldecane	5.27-5.30
1087	1091	p-cymenene	0.21-0.22
1100	1100	undecane	6.21-6.26
1261	1261	2-methyldodecane	1.30-1.32
1299	1300	tridecane	0.60-0.61
1371	1374	α-copaene	0.33
1406	1411	2-epi-β-funebrene	0.35
1413	1417	trans-β-caryophyllene	1.69-1.71
1474	1478	γ-muurolene	0.88-0.89
1499	1500	α-muurolene	0.30-0.31
1512	1513	γ-cadinene	0.52-0.53
1521	1522	trans-calamenene	0.44
1571	1577	spathulenol	0.23-0.28
1576	1582	caryophyllene oxide	1.95-1.99
1837	-	unknown	0.17-0.19
1895	-	unknown	0.16-0.17
1956	-	unknown	0.29

Observed KI	Adams KI	Compound name (from Bulgaria)	Min-Max (%)
743	745	4-methyl-2-Pentanol	0.26-0.27
861	865	2-Methyloctane	9.03-9.23
900	900	n-Nonane	1.61-1.97
926	924	α-Thujene	0.55-0.57
935	932	α-Pinene	8.63-8.77
947	946	Camphene	0.467-0.48
968	969	Sabinene	0.235
970	972	1-Octen-3-one	1.86-2.40
973	974	β-Pinene	3.01-3.08
1004	1002	α-Phellandrene	0.60-0.62
1015	1014	α-Terpinene	0.31-0.32
1022	1020	p-Cymene	0.64-0.65
1023	1024	D-Limonene	3.35-3.83
1025	1026	Eucalyptol	0.39-0.40
1034	1032	β-cis-Ocimene	0.30

1040	1044	β-trans-Ocimene	0.35-0.36
1044	1049	Isobutyl angelate	1.25-1.28
1055	1054	γ-Terpinene	0.70-0.72
1066	1063	n-Octanol	1.11-1.13
1085	1086	Terpinolene	0.35-0.36
1089	1087	2-Nonanone	0.45-0.46
1096	1095	β-Linalool	2.08-2.50
1120	1123	2-Methylbutyl-2-methylbutyrate	0.28
1141	1143	Isoamyl angelate	0.11
1149	1148	Isoamyl tiglate	1.10-1.08
1178	1174	1-Terpinen-4-ol	1.06-1.08
1199	1197	(2Z)-Octenol acetate	0.59-0.61
1203	1201	n-Decanal	0.10-0.09
1226	1227	Nerol	1.45-1.48
1232	1231	tetrahydro-Linalyl acetate	0.31-0.32
1247	1249	3-Methyl pentyl angelate	0.22-0.23
1291	1293	2-Undecanone	0.25-0.24
1301	1300	n-Tridecane	0.07
1357	1359	Neryl acetate	8.93-9.47
1366	1365	(2E)-Undecenol	0.17
1373	1374	α-Copaene	1.12-1.15
1400	1400	n-Tetradecane	0.18-0.19
1406	1405	Italicene	2.79-2.85
1409	1411	α-cis-Bergamotene	0.61-0.62
1419	1417	β-Caryophyllene	2.39-2.45
1420	1421	p-Cymen-7-ol acetate	1.61-1.64
1423	1422	Linalool butanoate	0.14-0.15
1429	1430	β-Copaene	0.37-0.38
1436	1437	α-Guaiene	2.72-2.97
1439	1440	(Z)-β-Farnesene	0.56-0.57
1444	1445	(2Z.6E)-Dodecadien-1-al	0.29-0.30
1450	1449	α-Himachalene	0.32-0.30
1470	1469	n-Dodecanol	0.51-0.52
1475	1474	Undecanal. dimethyl acetal	0.58-0.59
1481	1479	γ-Curcumene	0.77-0.79
1502	1500	β-Himachalene	7.34-7.82
1513	1514	β-Curcumene	2.46-2.52
1524	1522	δ-Cadinene	0.82-0.84
1539	1537	α-Cadinene	1.05-1.40
1546	1544	α-Calacorene	0.61-0.63
1550	1552	cis-Cadinene ether	0.56-0.57
1556	1557	trans-Cadinene ether	0.68-0.70
1560	1564	β-Calacorene	1.07-1.10
1575	1574	Germacrene D-4-ol	0.53-0.54
1576	1575	Pentyl salicylate	0.64-0.66
1578	1577	(-)-Spathulenol	0.54-0.55
1580	1582	Caryophyllene oxide	0.29-0.30
1592	1590	Globulol	1.80-1.96
1633	1630	γ-Eudesmol	2.39-2.45
1637	1638	tau.-Cadinol	0.21
1642	1640	tau.-Muurolol	1.34-1.36

1647	1649	β-Eudesmol	0.16
1650	1652	α-Eudesmol	2.11-2.16
1656	1655	Geranyl valerate	0.49
1665	1667	(6Z)-Pentadecen-2-one	0.25-0.26
1686	1685	Germacra-4(15).5.10(14)-trien-1α-ol	0.19
1700	1700	n-Heptadecane	0.18
1720	1722	(2Z.6E)-Farnesol	0.13
1743	1740	(2E.6E)-Farnesal	0.10
1765	1767	14-oxy-α-Muurolene	0.51-0.52
1778	1779	14-hydroxy-α-Muurolene	0.24-0.25
1784	1783	(2E.6E)-Methyl farnesoate	0.69-0.70
1791	1789	1-Octadecene	0.48

*** Kovats Index**

Table S6. Essential oil composition of *Hypericum sp.* from second experiment

Observed KI*	Adams KI	Name	HP1	HP2	Hcer	HR	Hcal. flos	Hcal. leaves
			Aver ± SD					
900	900	n-Nonane	0.35±0.02	0.50±0.01	nd	0.88±0.01	5.33±0.02	1.78±0.01
926	924	α-Thujene	0.13±0.01	0.04±	nd	nd	nd	nd
929	926	Ethyl 2-methyl pentanoate	nd	0.57±0.02	6.87±0.01	3.39±0.02	0.98±0.01	0.70±0.01
935	932	α-Pinene	6.76±0.30	6.41±0.22	nd	9.89±0.38	23.86±1.01	7.99±0.46
947	946	Camphene	nd	nd	nd	0.73±0.02	0.36±0.01	0.79±0.02
968	969	Sabinene	0.44±0.02	0.74±0.02	nd	0.95±0.01	0.23±0.01	0.13±0.01
973	974	β-Pinene	3.04±0.01	3.51±0.01	nd	16.43±1.25	29.37±2.017	20.62±2.028
985	988	β-Myrcene	2.52±0.02	3.00±0.05	nd	5.95±0.78	6.48±1.46	2.39±0.65
1004	1002	α-Phellandrene	nd	nd	nd	2.93±0.01	0.17±0.01	0.15±0.01
1015	1014	α-Terpinene	0.15±0.01	0.28±0.02	nd	1.91±0.01	0.38±0.01	0.65±0.03
1022	1020	p-Cymene	0.18±0.02	0.49±0.03	nd	0.99±0.01	0.09±0.00	0.10±0.02
1023	1024	D-Limonene	0.22±0.01	0.38±0.02	nd	3.75±0.11	9.74±1.01	14.44±2.05
1028	1026	β-Phellandrene	0.12±0.01	0.28±0.01	nd	1.40±0.02	0.11±0.02	0.46±0.04
1034	1032	cis-β-Ocimene	0.45±0.02	0.72±0.01	nd	8.69±0.02	0.37±0.02	3.92±0.02
1040	1044	trans-β-Ocimene	5.28±0.02	5.81±0.02	nd	12.88±0.02	0.61±0.01	0.65±0.01
1055	1054	γ-Terpinene	0.35±0.02	0.72±0.01	nd	3.31±0.03	0.70±0.02	1.21±0.02
1066	1063	n-Octanol	0.64±0.03	1.07±0.03	nd	0.15±0.01	0.50±0.02	0.14±0.02
1085	1086	Terpinolene	0.09±0.01	0.18±0.02	nd	1.20±0.02	1.10±0.03	1.25±0.04
1102	1100	n-Undecane	0.38±0.01	0.64±0.02	nd	1.33±0.1	nd	nd
1103	1102	Nonanal	0.09±0.01	0.18±0.02	nd	0.45±0.01	nd	nd
1178	1174	1-Terpinen-4-ol	0.11±0.01	0.16±0.02	nd	1.06±0.01	0.08±0.00	0.23±0.01
1265	1268	trans-2-decenol	0.13±0.01	0.32±0.02	nd	0.16±0.01	0.13±0.02	0.16±0.02
1270	1272	(3Z)-Nonenal dimethyl acetal	nd	nd	nd	0.67±0.02	0.53±0.02	0.28±0.02
1280	1277	Nonanal dimethyl acetal	nd	nd	nd	0.61±0.02	0.63±0.02	1.57±0.23
1292	1289	Thymol	nd	nd	8.31±1.32	nd	nd	nd
1347	1351	α-Cubebene	0.25±0.02	0.78±0.02	nd	1.41±0.02	0.21±0.02	nd
1351	1349	Thymol acetate	nd	nd	4.77±0.02	nd	nd	nd
1373	1374	α-Copaene	0.20±0.01	0.19±0.01	nd	0.25±0.01	0.14±0.01	0.32±0.01
1390	1388	β-Bourbonene	0.66±0.02	0.78±0.02	nd	0.08±0.00	0.39±0.02	0.59±0.02

1403	1399	Dodecanal	0.55±0.02	0.98±0.01	nd	2.25±0.10	0.16±0.01	0.10±0.01
1419	1417	β-Caryophyllene	16.08±0.02	6.20±0.02	nd	0.79±0.02	1.53±0.11	4.15±0.12
1436	1439	Aromadendrene	0.62±0.02	0.98±0.03	nd	0.42±0.01	0.22±0.03	0.14±0.02
1442	1440	β-Farnesene	4.05±0.02	6.03±0.76	nd	0.11±0.01	1.02±0.01	0.45±0.01
1453	1343	α-Humullene	1.80±0.01	1.68±0.01	nd	0.14±0.01	0.16±0.01	6.70±0.84
1484	1481	Germacrene D	12.87±1.49	16.08±0.94	nd	3.82±0.23	6.50±0.47	8.84±0.57
1502	1500	α-Muurolene	2.28±0.02	2.62±0.054	nd	0.57±0.05	0.40±0.02	3.13±0.12
1510	1513	γ-Cadinene	3.72±0.21	3.13±0.41	nd	0.35±0.02	2.19±0.02	0.54±0.03
1521	1522	δ-Cadinene	3.51±0.16	3.54±0.03	nd	0.15±0.03	0.96±0.02	1.55±0.02
1550	1553	Thymohydroquinone	nd	nd	3.63±0.12	nd	nd	0.31±0.02
1564	1561	trans-Nerolidol	2.25±0.01	3.45±0.01	nd	0.72±0.01	0.15±0.02	0.19±0.02
1579	1577	(-)-Spathulenol	3.95±0.01	3.50±0.1	nd	0.10±0.01	0.17±0.01	0.32±0.01
1580	1582	Caryophyllene oxide	5.12±0.13	3.35±0.01	nd	0.16±0.01	0.16±0.01	0.40±0.01
1633	1630	γ-Eudesmol	0.95±0.02	0.87±0.01	nd	0.09±0.00	0.12±0.00	0.40±0.02
1637	1638	tau.-Cadinol	2.03±0.01	3.36±0.01	nd	0.36±0.01	0.44±0.02	1.60±0.10
1642	1640	tau.-Muurolol	1.97±0.02	4.45±0.12	nd	0.48±0.01	0.13±0.01	0.64±0.01
1647	1649	α-Muurolol (Torreyol)	2.96±0.02	2.71±0.03	nd	0.47±0.01	0.56±0.02	5.66±0.42
1650	1652	α-Eudesmol	2.31±0.02	2.62±0.03	nd	0.11±0.01	0.22±0.02	0.16±0.01
1777	1779	α-14-hydroxy-Muurolene	2.30±0.14	2.40±0.01	nd	1.29±0.05	0.12±0.02	0.98±0.03
1871	1874	1-Hexadecanol	0.23±0.02	0.26±0.01	nd	0.23±0.01	0.12±0.01	nd
1900	1900	n-Nonadecane	0.19±0.01	0.27±0.02	nd	0.50±0.02	0.28±0.02	0.36±0.01
1955	1959	n-Hexadecanoic acid	2.51±0.04	0.67±0.02	36.48±1.45	nd	nd	nd
2101	2100	n-Heneicosane	0.28±0.02	0.10±0.01	0.13±0.01	0.22±0.02	0.35±0.01	0.37±0.02
2122	2118	3,7,11,15-Tetramethyl-2-hexadecen-1-ol	0.13±0.01	0.19±	28.49±0.02	0.09±0.02	0.12±	nd
2135	2132	(Z,Z)-9,12-octadecadienoic acid	nd	nd	3.45±0.02	nd	nd	nd
2144	2141	(Z,Z,Z)-9,12, 15-octadecatrienoic acid	0.85±0.02	0.12±0.01	4.03±0.01	nd	nd	nd
2302	2300	n-Tricosane	0.11±0.01	0.09±0.00	2.47±0.03	0.25±0.01	0.16±0.01	0.18±0.01
2501	2500	n-Pentacosane	0.14±0.01	0.18±0.02	nd	0.18±0.01	0.45±0.02	0.14±0.01
2803	2800	n-Octacosane	0.52±0.02	0.19±0.01	nd	0.68±0.02	0.20±0.01	0.17±0.02
2900	2900	n-Nonacosane	1.42±0.03	0.48±0.02	nd	2.45±0.02	0.25±0.01	0.20±0.01

*** Kovats Index**
